# Prolonged survival in advanced gallbladder cancer following tislelizumab combined with chemotherapy: Case report and literature review

**DOI:** 10.1097/MD.0000000000043592

**Published:** 2025-07-25

**Authors:** Da Ye, Zhiquan Qin, Peiyuan Yan, Qunjiang Wang, Jing Qu, Qihao Zhou

**Affiliations:** a Department of Medical Oncology, Cancer Center, Zhejiang Provincial People’s Hospital, Affiliated People’s Hospital, Hangzhou Medical College, Hangzhou, Zhejiang Province, China.

**Keywords:** advanced gallbladder cancer, case report, chemoimmunotherapy, immune checkpoint inhibitors, tislelizumab

## Abstract

**Rationale:**

Gallbladder cancer (GBC) is a highly aggressive cancer. When treated using standard chemotherapy, the median overall survival is <1 year. Immune checkpoint inhibitors such as pembrolizumab or durvalumab combined with chemotherapy show promise. However, those immune checkpoint inhibitors are very expensive. Tislelizumab may offer a more affordable alternative for advanced GBC.

**Patient concerns:**

We report on the case of a 70-year-old patient with GBC who, after experiencing disease progression following standard second-line chemotherapy, was excluded from participating in a clinical trial due to poor performance status.

**Diagnoses:**

The patient was diagnosed with stage IVB (TxN2M0) GBC.

**Interventions:**

He was treated with tislelizumab in combination with oxaliplatin and capecitabine.

**Outcomes:**

The patient had a progression-free survival of 7 months and overall survival of 16 months. The overall overall survival from the onset of the disease was 23 months.

**Lessons:**

The administration of tislelizumab improved survival in a patient with advanced gallbladder cancer. Tislelizumab emerged as a potential more cost-effective alternative option to pembrolizumab or durvalumab in our treatment strategy. These findings provide the basis for large-scale clinical trials to confirm the efficacy of tislelizumab for GBC.

## 1. Introduction

Gallbladder cancer (GBC) is a malignant biliary tract cancer (BTC) with a high incidence in Asia and Latin America.^[1]^ Since early diagnosis is challenging and systemic chemotherapy has limited efficacy, patients diagnosed with GBC generally have a poor prognosis, with most patients failing to survive 5-years after diagnosis.^[2]^ For patients with resectable disease, the BILCAP trial suggested that adjuvant capecitabine may improve overall survival (OS), although the primary endpoint in the intention-to-treat population was not met.^[3]^ However, studies have demonstrated that the median survival of patients with advanced unresectable GBC treated with standard chemotherapy is less than 1 year.^[4,5]^ Although first-line chemotherapy has been shown to be moderately effective in treating the disease, the use of second and third-line chemotherapy has exhibited a low success rate.^[6,7]^

The systemic treatment for BTC has evolved from conventional chemotherapy to integrated targeted and immunotherapeutic approaches. Although gemcitabine plus cisplatin remains the first-line treatment for GBC, new targeted therapies such as fibroblast growth factor receptor (FGFR) inhibitors (e.g., pemigatinib) and isocitrate dehydrogenase 1 (IDH1) inhibitors (e.g., ivosidenib) may have a role in treating GBC with specific genomic alterations.^[8,9]^ Immune checkpoint inhibitors (ICIs) have shown promising results in the management of GBC.^[10]^ As monotherapy, nivolumab, a programmed death-1 (PD-1) inhibitor, demonstrated efficacy in pretreated BTC patients, with an overall response rate (ORR) of 3.3% to 22%.^[9]^ The TOPAZ-1 trial demonstrated that adding durvalumab, a PD-L1 inhibitor, to standard chemotherapy (gemcitabine plus cisplatin [GEMCis]) significantly improved OS, progression-free survival (PFS), and ORR compared to chemotherapy alone in patients with advanced BTC.^[11]^ These results provided evidence for durvalumab combined with chemotherapy as a new first-line standard treatment. Similarly, in the KEYNOTE-996 trial, patients treated with pembrolizumab (a PD-1 inhibitor) combined with chemotherapy had an overall improvement in the OS when compared with a group of patients treated with a placebo.^[12]^ Recent first-line trials combining ICIs such as pembrolizumab or durvalumab with chemotherapy have reported median OS of approximately 12.7 to 12.8 months,^[11,12]^ while some combination regimens, like camrelizumab plus gemcitabine and oxaliplatin (GEMOX), have shown median OS up to 11.8 months.^[13]^ Emerging biomarkers such as the neutrophil-to-eosinophil ratio and the cachexia index offer valuable prognostic insights and could be used to predict response to systemic therapy.^[14,15]^ However, the high cost of novel therapies may still limit patient access to these treatments.

Tislelizumab (BGB-A317), another PD-1 inhibitor, may provide a cheaper alternative to Pembrolizumab or Durvalumab. Studies have shown a good response to tislelizumab in various solid tumors.^[16]^ Two separate studies documented the efficacy of tislelizumab plus Lenvatinib for elderly patients with advanced GBC and intrahepatic cholangiocarcinoma as a first-line treatment.^[17,18]^

In this case study, we report on the first use of tislelizumab in combination with chemotherapy to treat a patient diagnosed with stage IV B GBC with low PD-L1 expression. Following disease progression after first-line treatment with GEMOX, the patient was treated with 2 cycles of second-line nab-paclitaxel and gemcitabine. However, since the patient had a poor response to treatment, he was subsequently treated with tislelizumab combined with oxaliplatin and capecitabine. The patient achieved a partial response with a PFS of 7 months and an OS of 16 months, which is higher than the OS reported by trials using pembrolizumab or durvalumab.

## 2. Case presentation

**Figure 1.**
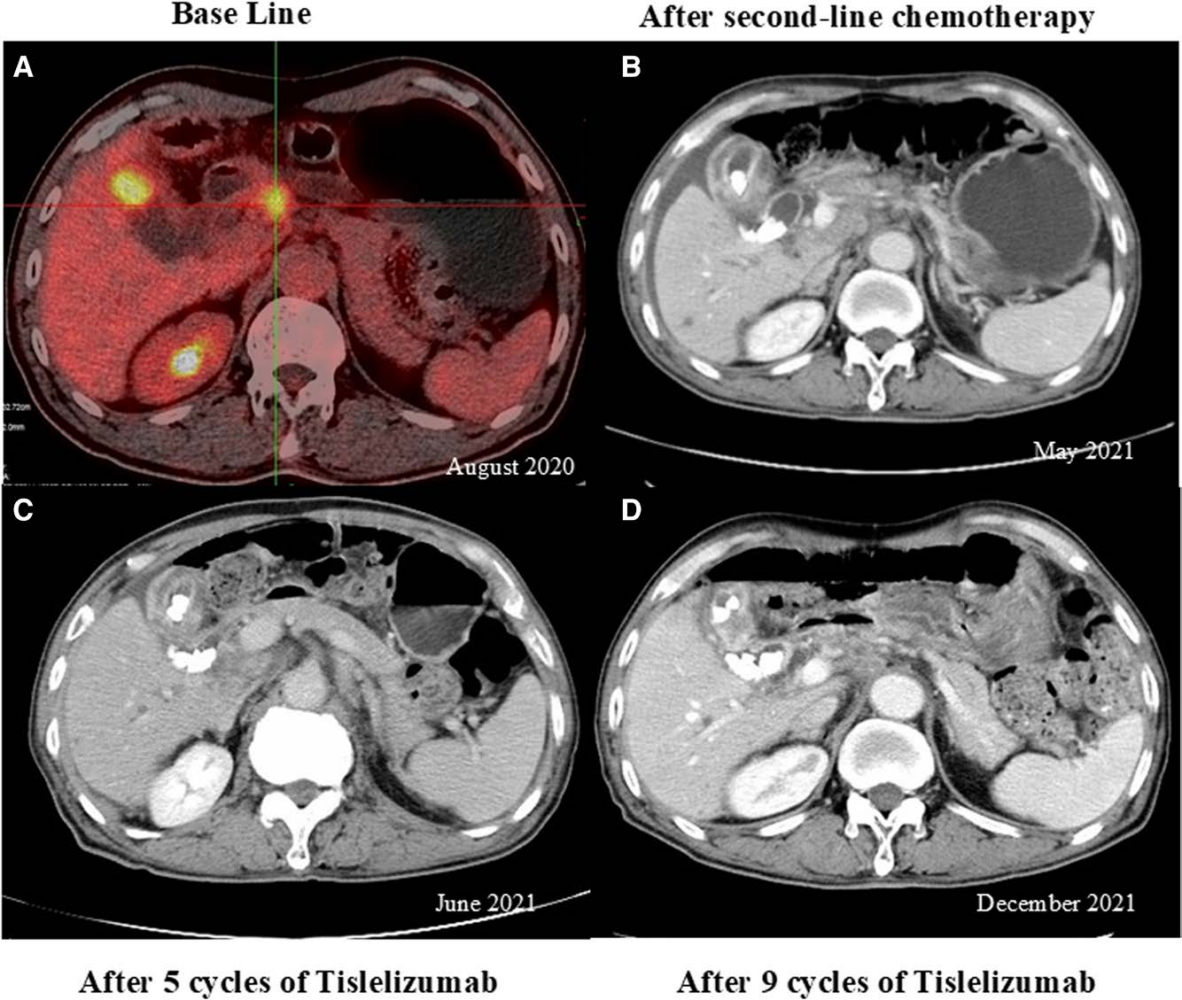
Radiological images of the patient. (A) Pretreatment PET/CT showing enlarged lymph nodes and a thickened gallbladder wall. (B) CT acquired after second-line chemotherapy showing a gallbladder mass and disease progression to the abdominal lymph nodes. (C) CT acquired after 5 tislelizumab cycles showing a reduction in the size of several lymph nodes. (D) CT acquired after 9 tislelizumab cycles showing lymph node enlargement indicating disease progression. PET/CT = tomography/computed tomography.

**Figure 2.**
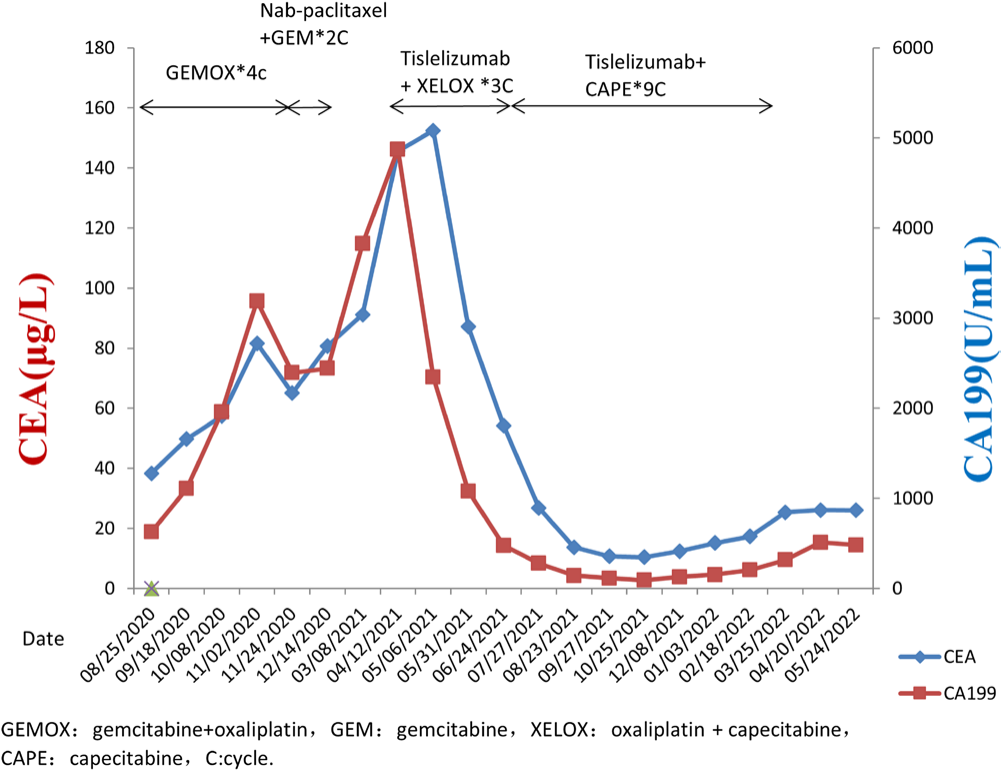
Changes in the CEA and CA19-9 markers during treatment. CEA = carcinoembryonic antigen.

A 70-year-old male with a smoking history presented at our hospital on August 25, 2020, with an abdominal mass. Positron emission tomography/computed tomography (CT) imaging revealed thickening of the gallbladder, along with multiple enlarged abdominal lymph nodes (Fig. 1A). Laboratory assessments indicated elevated levels of carcinoembryonic antigen (CEA) at 38.3 μg/L (normal CEA ≤ 5 μg/L) and serum carbohydrate antigen 19-9 at 626.1 U/mL (normal CA ≤ 37.0 U/mL). Pathological and immunohistochemical examination of the gallbladder *via* transmural puncture revealed poorly differentiated adenocarcinoma. The patient had a low PD-L1 expression with a combined positive score of <1 (clone 22C3; Dako, Glostrup, Denmark). According to the American Joint Committee on Cancer staging manual, the patient was diagnosed with stage IVB (TxN2M0) GBC. Subsequently, he was treated with 4 chemotherapy cycles of gemcitabine (Hansoh Pharma Co., Ltd, Jiangsu, China) (1.6 g each at days 1 and 5) and oxaliplatin (Hengrui Pharmaceuticals Co., Ltd. Jiangsu, China) (150 mg at day 1). Following this treatment, laboratory tests showed increased levels of CEA (80.7 μg/L) and CA19-9 (2442.0 U/mL). Therefore, the patient was treated with an additional 2 cycles of nab-paclitaxel (CSPC, OUYI Pharmaceutical Co., Ltd, Shijiazhuang, Hebei, China) (200 mg per cycle on days 1 and 5) and gemcitabine (1.6 g per cycle on days 1 and 5). Nonetheless, the CEA and CA19-9 levels continued to rise (CEA: 91.2 μg/L and CA19-9: 3828 U/mL), indicating disease progression. Two months later, the patient presented with severe weight loss, weakness, and abdominal distension, prompting hospitalization. Laboratory assessments indicated a further elevation in CEA (145.6 μg/L) and CA19-9 (4872 U/mL). The patient could not be enrolled in a clinical trial due to poor performance status (PS). Subsequently, he was treated with 3 cycles of tislelizumab (Baize’an; BeiGene Ltd., Beijing, China) (200 mg at day 1) in combination with oxaliplatin and capecitabine (Qilu Pharmaceutical Co., Ltd, Shandong, China) (1.5 g twice daily for 14 consecutive days). After completion of the third cycle of chemotherapy, the laboratory test results showed a significant decline in the CEA (54.2 μg/L) and CA19-9 (477.1 U/mL). In addition, an abdominal CT revealed a reduction in the metastatic multiple lymph nodes (Fig. 1C), indicating a partial response to treatment. Due to dose-limiting toxicity associated with oxaliplatin, the patient received 9 cycles of tislelizumab plus capecitabine. Throughout the treatment, the patient experienced minor episodes of nausea and vomiting. A gradual increase in the CEA and CA19-9 levels was observed (Fig. 2), accompanied by a slow increase in metastatic lymph nodes and CT scans. Unfortunately, the patient passed away in his hometown in August 2022. Following the administration of the chemoimmunotherapy, the patient had a PFS of 7 months and an OS of 16 months. The total OS from disease onset was 23 months.

### 2.1. Patient and family perspective (obtained from immediate family)

The patient’s family described profound distress after the patient was diagnosed with stage IV GBC. When standard therapies failed and poor performance excluded him from trials, tislelizumab’s affordability compared to other immunotherapies was decisive. During treatment, they observed manageable side effects and a brief period of improved physical and mental status when tumor markers dropped, offering cherished relief. Though the outcome was heartbreaking, the family emphasized that the extended survival achieved through this accessible option was invaluable. They stress that the affordability of therapeutic drugs matters deeply for families facing advanced cancer.

## 3. Discussion and literature review

GBC is an invasive adenocarcinoma of the gallbladder. Thailand and China have a high incidence rate compared to other countries worldwide.^[19]^ Most GBC cases are diagnosed at advanced, unresectable stages, making systemic chemotherapy the mainstay of treatment.^[20]^ Table 1 presents recent trials on advanced BTC, which include some patients with GBC.^[4,5,7,11–13,21–23]^ The ABC-02 trial of 410 patients established gemcitabine plus cisplatin (GemCis) as the first-line standard of care for advanced BTC patients, with a median OS of 11.7 months, compared to 8.1 months with Gemcitabine alone.^[5]^ In another trial, modified GEMOX therapy in 81 unresectable GBC patients achieved significantly better OS and PFS when compared to supportive care and fluorouracil monotherapy.^[4]^ Despite these improvements, the median OS for advanced GBC patients receiving chemotherapy remains <1 year. Second-line treatment options are limited. In a retrospective study, Caparica et al^[6]^ showed that the use of fluorouracil and irinotecan as second-line therapy resulted in a PFS of 1.7 months and a median OS of 5 months. A systematic review by Lamarca et al,^[7]^ involving 761 advanced BTC or GBC patients, further highlighted the lack of evidence for specific second-line regimens, emphasizing the need for prospective randomized studies to guide treatment decisions.

**Table 1:** Clinical trials of advanced biliary tract cancer.

References	Trial	Patients	Treatment line	Procedures	Results
Valle et al^[5]^	Randomized, controlled, phase 3 trial clinical trials. (NCT00262769).	410 BTC patients(149/36% BGC patients)	First-line	GemCis group. Gemcitabine-only group	mOS:11.7 VS 8.1 months; mPFS: 8 vs 5 months
Sharma et al^[4]^	Randomized, controlled, open-label, single-center study conducted.	81 GBC patients.	First-line or first-line after recurrence.	A group: best supportive care; B group: fluorouracil and folinic acid; C group: modified GEMOX.	mOS:4.5 vs 4.6 vs 9.5 months; mPFS: 2.8 vs 3.5 vs 8.5 months.
Shroff et al^[21]^	Open-label, single-arm, phase 2 clinical trial. (NCT02392637)	62 BTC patients (13/22% BGC patients)	First-line	GemCis and nab-paclitaxel	mPFS: 11.8 months, mOS: 19.2 months.
Ueno et al^[22]^	Non-randomized, multicentre, open-label, phase 1 study. (JapicCTI-153098)	60 BTC patients (20/33%BGC patients)	First-line	Nivolumab monotherapy group. Nivolumab plus GemCis group.	mOS: 5.2 vs 15.4 months; mPFS: 1.4 vs 4.2 months.
Chen et al^[13]^	Single- arm, open- label, phase II study. (NCT03486678)	38 BTC patients	First-line	Camrelizumab plus Gemox	mPFS: 6.1 months; mOS: 11.8 months.
Piha-Paul et al^[23]^	KEYNOTE-158 (NCT02628067; phase 2)	104 BTC patients	Disease progression after ≥ 1 prior standard therapy or were not candidates for any other standard therapy.	Pembrolizumab	mOS: 7.4 months; mPFS: 2 months.
Piha-Paul et al^[23]^	KEYNOTE-028 (NCT02054806; phase 1b)	PD-L1 positivity 24 BTC patients	Disease progression after ≥ 1 prior standard therapy or were not candidates for any other standard therapy.	Pembrolizumab	mOS: 5.7 months; mPFS: 1.8 months.
Oh et al^[11]^	TOPAZ-1 trial. Double-blind, placebo-controlled, phase 3 study. (NCT03875235)	685 BTC patients(171/25%BGC patients)	First-line or first-line after recurrence	Durvalumab or placebo in combination with GemCis	mPFS: 7.2 vs 5.7 months; mOS:12.8 vs 11.5 months.
Kelley et al^[12]^	KEYNOTE-966 study. Randomized, double-blind, placebo-controlled, phase 3 trial. (NCT04003636)	1069 BTC patients (233/22%GBC patients)	First-line or first-line after recurrence	Pembrolizumab or placebo with GemCis	mOS:12.7 vs 10.9 months; mPFS:6.5 vs 5.6 months.
Caparica et al^[6]^	Single-institution, retrospective cohort study	12 BTC patients (1/8%GBC patient)	Second-line therapy	FOLFIRI	mPFS: 1.7 months; mOS: 5 months.
Lamarca et al^[7]^	Systematic literature review	585 studies, 761 patients.	Second-line	–	mOS: 7.2 months; mPFS: 3.2 months.

BGC = gallbladder cancer, BTC = biliary tract cancer, FOLFIRI = fluorouracil plus irinotecan, GBC = gallbladder cancer, GemCis = cisplatin plus gemcitabine, GEMOX = gemcitabine plus oxaliplatin, mOS = median overall survival, PFS = median progression-free survival.

In our case, the patient initially received GEMOX as a first-line treatment but failed to show any treatment response after 4 cycles. The elevated CEA and CA19-9 levels and abdominal CT scan confirmed disease progression. Subsequently, nab-paclitaxel and gemcitabine were administered as a second-line treatment, but the patient was lost to follow-up. Upon rehospitalization, disease progression was evident. In a similar context, the single-arm Phase II clinical trial conducted by Shroff et al demonstrated that, compared with historical controls treated with GemCis alone, the combination of nab-paclitaxel and GemCis prolonged PFS and OS.^[21]^

ICIs are revolutionizing the treatment for patients with advanced cancer, including BTC. The KEYNOTE-028 and KEYNOTE-158 trials showed that pembrolizumab resulted in an ORR for survival of 5.8% in patients with advanced solid GBC.^[23]^ As a result, pembrolizumab is now recommended as a first-line treatment for patients with specific tumor profiles characterized by deoxyribonucleic acid mismatch repair deficiency, microsatellite instability-high tumors, and a tumor mutational burden above 10.

Combining ICIs with chemotherapy as a first-line strategy has shown promising results. Studies involving nivolumab in combination with GemCis and camrelizumab with GEMOX have significantly increased the median OS and PFS in BTC patients^[13,22]^ The TOPAZ-1, a double-blind, placebo-controlled phase III trial, enrolled 685 patients, including 171 with unresectable or metastatic BTC or recurrent GBC. These patients received either durvalumab or a placebo in conjunction with GemCis. Compared to the placebo, durvalumab resulted in a marked improvement in both the median PFS (7.2 months vs 5.7 months) and OS (12.8 months vs 11.5 months).^[11]^ These findings suggest that combining ICIs with chemotherapy may improve the outcomes of patients with advanced GBC. The KEYNOTE-966 phase III trial demonstrated that patients treated with pembrolizumab in combination with GemCis showed a significant improvement in both OS (12.7 vs 10.9 months) and PFS (6.5 vs 5.6 months) compared to those receiving placebo.^[12]^ Additionally, a case report has also shown that a stage IVB GBC patient achieved a durable complete response of more than 32 months after being treated with a primary tumor resection followed by a combination tegafur/gimeracil/oteracil and pembrolizumab.^[24]^

In our case, the patient was treated with ICIs in combination with chemotherapy after experiencing disease progression following second-line chemotherapy treatment. The patient had a partial response, a 7-month PFS, and a 16-month OS after chemoimmunotherapy. These outcomes underscore the potential of chemoimmunotherapy as a viable therapeutic option in advanced GBC.

Tislelizumab has a unique structure that has been carefully engineered to inhibit the binding of PD-1 to PD-L1 and minimize its binding to the Fcγ receptors. This ICI was found to be an effective treatment for various solid tumors.^[16]^ However, its application in BTC remains limited. Notably, a case series by Wang et al demonstrated a positive response in 4 elderly patients with advanced GBC treated with tislelizumab in combination with lenvatinib and chemotherapy.^[18]^ In a case report by Ding et al,^[17]^ a patient diagnosed with intrahepatic cholangiocarcinoma underwent 4 cycles of gemcitabine, cisplatin, lenvatinib, and tislelizumab. This patient achieved a partial response and underwent subsequent surgery, after which he continued the immunotherapy for 1 month without recurrence. Our case report describes a stage IVB GBC patient with localized primary tumor and lymph node metastasis who, after disease progression on standard therapies, received tislelizumab combined with oxaliplatin and capecitabine and achieved a partial response. While PD-L1 expression is commonly used to predict immunotherapy response, it is not always a good predictor of treatment response since its expression levels can be affected by variability in staining antibodies and expression patterns.^[25]^ Studies have shown that the expression levels of PD-L1 in BTC can range between 9.1% to 72.2% of BTCs.^[20]^ Although some studies suggest that ICIs are only effective in PD-L1-positive patients,^[13,23]^ our case represents an exception as the patient still benefited from immunotherapy despite negative PD-L1 expression. These findings suggest that PD-L1 may not be the perfect predictor of immunotherapy response in GBC.

The standard treatment for advanced GBC primarily relies on chemotherapy, with patients often experiencing a median survival of less than a year. Promising developments have emerged by combining chemotherapy with immunotherapy, specifically utilizing agents like pembrolizumab or durvalumab. The preliminary clinical trial results of pembrolizumab to treat GBC have shown an OS of 12.7 months and PFS of 6.5 months.^[12]^ On the other hand, durvalumab resulted in an OS of 12.8 months, and the PFS was 7.2 months.^[11]^ In our specific case, despite the patient presenting with poor performance status, we opted to use tislelizumab, following an inadequate response to second-line chemotherapy. At that time, the clinical trial results of durvalumab and pembrolizumab had not yet been published. Following the administration of tislelizumab, our patient achieved a PFS of 7 months and an OS of 16 months. The overall OS from the onset of the disease was 23 months. Apart from the role of tislelizumab as a first-line therapy, these findings further support the use of this treatment for advanced GBC, especially for patients with a more favorable performance status. Moreover, tislelizumab emerged as a valuable, more cost-effective alternative to pembrolizumab or durvalumab in our treatment strategy.

This case report highlights critical knowledge gaps and future directions in the management of advanced GBC, such as the urgent need to develop robust predictive biomarkers beyond PD-L1. Although PD-L1 expression remains a standard biomarker, its predictive utility in BTCs is inconsistent, as exemplified by our PD-L1-negative patient’s favorable response to tislelizumab. Further large-scale genomic and immunophenotypic profiling studies are warranted to identify more reliable biomarkers, such as TMB, specific mutational signatures including IDH1 and FGFR2 alterations, or novel immune microenvironment signatures that can more accurately stratify patients likely to benefit from ICIs like tislelizumab. Additionally, the optimal sequencing of therapeutic regimens remains undefined. Our patient demonstrated notable survival benefits following failure of standard treatment. These findings suggest a potential role for tislelizumab-based chemoimmunotherapy as a second or third-line treatment for GBC patients. However, more research is required to evaluate the role of this treatment for vulnerable populations, such as elderly patients with deteriorating performance status. To address these challenges, focused clinical trials and robust real-world evidence studies are imperative to inform tailored therapeutic strategies.

Looking ahead, we envision significant changes in the management of GBC over the next 5 years. We anticipate a shift towards more personalized, biomarker-driven approaches. Combinations of ICIs with targeted therapies (e.g., FGFR inhibitors, IDH1 inhibitors) or novel agents like antibody-drug conjugates will likely become central, moving beyond chemotherapy backbones. Trials exploring these combinations, potentially including tislelizumab, are already emerging and will define new standards. Furthermore, research will focus on new strategies aimed at overcoming primary and acquired resistance to immunotherapy, such as targeting of alternative immune checkpoints (e.g., LAG-3, TIGIT) or modulation of the immunosuppressive tumor microenvironment. Real-world data platforms will become increasingly important to complement clinical trials, providing insights into long-term outcomes, optimal sequencing, and effectiveness in diverse patient populations outside strict trial criteria. Cost-effectiveness will remain a critical consideration globally, potentially enhancing the role of agents like tislelizumab if their clinical benefit is confirmed alongside their affordability.

This report has several limitations that have to be acknowledged. The patient’s two-month loss to follow-up after completing 2 cycles of second-line treatment could have resulted in a loss in the reporting of critical changes in disease status or treatment response, and thus reduces the reliability of outcome assessment. The observed discordance between tumor marker fluctuations (CEA and CA19-9) and imaging findings likely reflects tumor heterogeneity or differential responses among tumor clones, highlighting the need for a multimodal approach to treatment assessment. Additionally, the patient’s advanced age, poor baseline performance status, and cumulative toxicity from prior therapies represent significant confounding factors that may have influenced treatment tolerance, response, and survival. Therefore, the results should be interpreted with caution within this complex clinical context. Despite these limitations, our case suggests that tislelizumab provides similar outcomes to current standard ICIs, such as durvalumab and pembrolizumab, for patients with advanced GBC and may provide an alternative, more affordable option.

## 4. Conclusion

In this case study, we reported on an elderly male patient with GBC who failed both first- and second-line treatments. He received tislelizumab combined with chemotherapy and achieved a partial response and prolonged OS of 16 months post-immunotherapy and total OS of 23 months, which is a notable outcome for a patient with advanced PD-L1 negative GBC who had disease progression after first-line chemotherapy treatment and overall poor performance status. In line with recent trials, this case further highlights the potential benefits of using ICI such as tislelizumab in the management of advanced GBC patients. However, further large-scale clinical trials are required to confirm the efficacy and define the optimal role of tislelizumab in the management of advanced GBC.

## Acknowledgments

We thank the patient’s family for providing the necessary medical history required for this case report. We acknowledge TopEdit LLC for the linguistic editing and proofreading during the preparation of this manuscript.

## Author contributions

**Conceptualization:** Zhiquan Qin, Qihao Zhou.

**Data curation:** Da Ye, Qunjiang Wang, Jing Qu.

**Formal analysis:** Da Ye, Qihao Zhou.

**Investigation:** Peiyuan Yan, Jing Qu.

**Methodology:** Zhiquan Qin, Qihao Zhou.

**Writing – original draft:** Da Ye, Qihao Zhou.

**Writing – review & editing:** Qihao Zhou.
